# A plastid-targeted heat shock cognate 70-kDa protein confers osmotic stress tolerance by enhancing ROS scavenging capability

**DOI:** 10.3389/fpls.2022.1012145

**Published:** 2022-10-05

**Authors:** Feng Ding, Fan Li, Binglei Zhang

**Affiliations:** ^1^ State Key Laboratory of Hybrid Rice, College of Life Sciences, Wuhan University, Wuhan, China; ^2^ School of Landscape and Ecological Engineering, Hebei University of Engineering, Handan, China

**Keywords:** osmotic stress, ROS accumulation, antioxidant enzymes, cpHSC70-1, *Arabidopsis thaliana*

## Abstract

Osmotic stress severely affects plant growth and development, resulting in massive loss of crop quality and quantity worldwide. The 70-kDa heat shock proteins (HSP70s) are highly conserved molecular chaperones that play essential roles in cellular processes including abiotic stress responses. However, whether and how plastid-targeted heat shock cognate 70 kDa protein (cpHSC70-1) participates in plant osmotic stress response remain elusive. Here, we report that the expression of *cpHSC70-1* is significantly induced upon osmotic stress treatment. Phenotypic analyses reveal that the plants with *cpHSC70-1* deficiency are sensitive to osmotic stress and the plants overexpressing *cpHSC70-1* exhibit enhanced tolerance to osmotic stress. Consistently, the expression of the stress-responsive genes is lower in *cphsc70-1* mutant but higher in *35S:: cpHSC70-1* lines than that in wild-type plants when challenged with osmotic stress. Further, the *cphsc70-1* plants have less APX and SOD activity, and thus more ROS accumulation than the wild type when treated with mannitol, but the opposite is observed in the overexpression lines. Overall, our data reveal that *cpHSC70-1* is induced and functions positively in plant response to osmotic stress by promoting the expression of the stress-responsive genes and reducing ROS accumulation.

## Introduction

Environmental stresses such as drought, cold, and salinity can alter water availability by changes in solute concentrations (i.e., inorganic cations, sugars, anions, and salts) and impose osmotic stress on plants, which affects cell membrane integrity, photosynthetic capacity, and osmotic regulation, leading to severe restrictions on plant growth and development (Zhao et al., 2020). Osmotic stress reduces the water uptake of plants, bringing about not only ionic stress but also oxidative damages caused by overaccumulated reactive oxygen species (ROS) including hydroperoxide (H_2_O_2_), superoxide anion $\left(O_{2}^{•-}\right)$ , and hydroxyl radicals (•OH) (Wenjing et al., 2020; Zhao et al., 2020; Wang et al., 2021b; Zhang et al., 2021). These ROS can affect proteins, lipids, and nucleic acids if accumulated over a certain threshold, leading to cell damage and death (Yuan et al., 2017; Nadarajah, 2020). To overcome this issue, plants have employed complex antioxidant defense mechanisms to protect plants from osmotic stress-induced oxidative damage by scavenging ROS and maintaining the balance of ROS production (Hasanuzzaman et al., 2021).

The antioxidant defense system includes a series of enzymes such as catalase (CAT), superoxide dismutase (SOD), ascorbate peroxidase (APX), and non-enzymatic antioxidants (α-tocopherol, glutathione, β-carotene, ascorbate). They work in coordination to maintain ROS homeostasis by scavenging stress-induced excess ROS in plant cells (Waszczak et al., 2018; Sánchez-McSweeney et al., 2021). For instance, SOD can disproportionate O^2-^ to H_2_O_2_, and then H_2_O_2_ is further detoxified into H_2_O by APX with the assistance of ascorbate in the chloroplast (Uzilday et al., 2014) and thus overexpressing *SOD* and *APX* can improve the plant’s tolerance under salt stress (Shafi et al., 2019). CATs, the enzymes directly degrading H_2_O_2_ without assistance of any reducing equivalent, are also necessary for scavenging ROS in plants under various stressed conditions (Hasanuzzaman et al., 2020). It is reported that the plants with *CAT3* mutation accumulate higher H_2_O_2_ and are hypersensitive to water deprivation, but the plants overexpressing *CAT3* have less H_2_O_2_ and are more tolerant to drought stress compared with the wild type (Zou et al., 2015). Similarly, the disruption of *CAT2* causes excess H_2_O_2_ accumulation and reduces plant tolerance to high salinity (Bueso et al., 2007). Further, several factors have been indicated to regulate antioxidant enzymes in the stressed plants. For example, the zinc finger protein Zat12 involved in plant response to oxidative stress by promoting *APX1* expression and *Zat12*-deficient plants are more sensitive to H_2_O_2_ application than wild-type plants (Rizhsky et al., 2004). Overexpression of *AtbHLH112* increases salt and drought tolerance by promoting SOD activity to improve ROS scavenging ability (Liu et al., 2015). A recent report documented that leucine aminopeptidase 2 (LAP2), as a collaborator of CAT2, confers Arabidopsis plants increased osmotic stress tolerance possibly through maintaining CAT2 protein stability (Zhang et al., 2021). In addition, peroxisome-localized small heat shock protein Hsp17.6CII activates catalase by interacting with CAT2 and thus confers alkaline and salt stress tolerance in plants (Li et al., 2017). MeHSP90.9-silenced plants have repressed *CAT1* expression, reduced CAT activity, and higher H_2_O_2_, resulting in more sensitivity to drought stress (Wei et al., 2020). These reports reveal that modulations of antioxidant enzymes play a vital role in regulating plant abiotic stress tolerance.

Heat shock proteins (HSPs) are a diverse group of multifamily proteins, functioning in adverse stimuli by preventing protein misfolding, reducing the aggregation of denatured proteins, and maintaining protein structural stability (Sable et al., 2018; Qi et al., 2019). Based on their apparent molecular weight, amino acid sequence homology, and functions, HSPs have been classified into HSP100s, HSP90s, HSP70s, HSP60s, and small heat shock protein (sHSP) families (Mayer and Bukau, 2005). Of all HSPs, HSP70 superfamily members are the most abundant, highly conserved, and well-characterized group of molecular chaperones in all organisms from prokaryotes to eukaryotes (Xu et al., 2020; Li et al., 2021). Structurally, HSP70s are identified by three distinct domains: a 45-kDa N-terminal ATPase, a 15-kDa β-sandwich domain, and a 10-kDa C-terminal α-helical domain (Zhu et al., 1996; Tang et al., 2016). Functionally, while HSP70s assist cellular machinery in regulating protein degradation and verifying proteins quality under normal conditions (Bukau et al., 2006; Su and Li, 2008; Hartl et al., 2011), they facilitate denatured protein refold, prevent denatured proteins from aggregating, and dissolve or degrade protein aggregates during stress (Wang et al., 2004; Lee and Tsai, 2005). In Arabidopsis, 18 HSP70s have been identified, and they are divided into four subclasses based on their subcellular localization: cytosol/nucleus, mitochondria, endoplasmic reticulum (ER), and plastids (Wu et al., 1994; Lin et al., 2001). The cytosolic/nuclear HSP70s mainly function in plant development, signaling pathways, abiotic stresses including drought, salinity, and high temperature, and biotic stresses such as virus infection (Jungkunz et al., 2011; Leng et al., 2017). The mitochondria-localized mtHSC70-1 and mtHSC70-2 are required for the mitochondrial Fe–S cluster assembly and aid in the translocation of precursor proteins to mitochondria as part of the translocon (Zhang and Glaser, 2002; Leaden et al., 2014). Early reports showed that overexpression of the rice (*Oryza sativa*) *mtHSC70* inhibited heat- and H_2_O_2_-induced cell death in protoplasts through reducing reactive oxygen species (ROS) generation and sustaining mitochondrial membrane potential (Qi et al., 2011). A recent study showed that *mtHSC70-1* mutation resulted in severe embryo defects (Li et al., 2021). As ER-localized HSP70s, immunoglobulin-binding proteins (BiPs) play an important role in male and female gametophyte development and unfolded protein responses (Srivastava et al., 2013; Maruyama et al., 2014). For plastid-localized HSP70s in Arabidopsis, two cpHSC70s (cpHSC70-1 and cpHSC70-2) are identified to be essential for maintaining chloroplast structure and functions (Sung et al., 2001; Latijnhouwers et al., 2010). While *cphsc70-1* mutant plants exhibit variegated cotyledons, slow growth, deformed leaves, and impaired root growth under normal growth conditions, the stressed *cphsc70-1* mutant plants are more sensitive to high temperature and drought stress (Su and Li, 2008; Latijnhouwers et al., 2010). However, whether and how cpHSC70-1 participates in plant response to osmotic stress remains unknown.

In this study, we report that *cpHSC70-1* plays important roles in plant tolerance to osmotic stress. When challenged with osmotic stress, the expression of *cpHSC70-1* is promoted in plants. Moreover, the knockout of *cpHSC70-1* has enhanced sensitivity to osmotic stress with lower APX and SOD activities and increased ROS accumulation. Overexpression of *cpHSC70-1* in wild type improves tolerance of transgenic Arabidopsis to osmotic stress, with a higher expression of genes encoding antioxidant enzymes and decreased ROS accumulation. Taken together, these results show that osmotic stress-induced *cpHSC70-1* functions necessarily in the stress tolerance by modulating ROS scavenging capacity in Arabidopsis.

## Materials and methods

### Plant materials and growth conditions

The Columbia-0 (Col-0) *Arabidopsis thaliana* ecotype was employed in the present study. The mutants *cphsc70-1* (Salk_140810) and *cphsc70-2* (Salk_095715) were previously reported (Su and Li, 2008). Arabidopsis seeds were surface sterilized with 5% (w/v) bleach for 5 min, rinsed three times with sterile water, stored to 4°C for 3 days, and then grown on 1/2 strength MS (Murashige and Skoog) medium (pH 5.8) containing 1% (w/v) agar and 1% (w/v) sucrose, and plants were grown at 23°C with 16-h light (100 μmol m^–2^ s^–1^ illumination)/8-h dark conditions. For osmotic stress treatment, the corresponding seedlings were planted on 1/2 MS medium supplemented with or without 300 mM mannitol (Beijing Dingguo Changsheng Biotechnology Co., Ltd., DH190-2) for 5 days, and then the fresh weight and root length were determined and analyzed.

### Plasmid construction and plant transformation

The full-length coding sequences (CDS) of *cpHSC70-1* (AT4G24280), *cpHSC70-2* (AT5G49910), and the promoter (2 kb) of *cpHSC70-1* were amplified using PCR. The resulting fragments (*cpHSC70-1* and *cpHSC70-2*) were cloned into the pCAMBIA1300S vector and verified by sequencing. *cpHSC70-1* was also cloned into pCAMBIA1300 driven by the *cpHSC70-1* promoter. The resultant plasmids were transformed into Col-0 or the homozygous *cphsc70-1* mutant using *Agrobacterium tumefaciens* strain pGV3101 and the floral dip method. The primer sequences are listed in **Supplemental Table 1**.

### RNA extraction and quantitative reverse transcription PCR

The RNA was extracted as previously described (Ding et al., 2022). Briefly, plants were thoroughly ground with liquid nitrogen, and TRIzol reagent (Invitrogen) was used to extract the total RNA by following the manufacturer’s instructions, and RQ1 RNase-free DNase I (Promega) was employed to remove the contaminated DNA. RNA reverse transcription was performed with ReverTra Ace kit (TOYOBO) according to the manufacturer’s instructions. qPCR was performed using a Bio-Rad CFX96 and SYBR Green I dye (Invitrogen) with the program of 95°C for 3 min, 35 cycles of 95°C for 10 s, and 60°C for 45 s, followed by 5 min incubation at 95°C. *ACTIN2* (AT3G18780) was used as the reference gene, and all experiments included three independent biological replicates and three technical repetitions. Primer sequences are shown in **Supplemental Table 1**.

### Seed germination and cotyledon expansion statistics

Germination was determined in the same way as before (Ding et al., 2022). Briefly, the seeds were planted on 1/2 MS medium with or without 300 mM mannitol. The radicle’s appearance served as a gauge for seed germination. The percentages of germinated seeds were counted at the specified times. For each seed germination experiment, at least 60 seeds of each genotype were used, and experiments were conducted three times. On the fifth day, the percentages of cotyledon expansion per plant was scored.

### 3,3-Diaminobenzidine and nitroblue tetrazolium staining

As detailed earlier (Yu et al., 2019; Luo et al., 2021), the 7-day-old seedlings grown on 1/2 MS medium were transferred to mannitol (300 mM) containing 1/2 MS medium for 48 h, then the seedlings were stained with 3,3-diaminobenzidine (DAB) or nitroblue tetrazolium (NBT) to determine H_2_O_2_ or superoxide anion accumulation. For DAB staining, seedlings were incubated for 8 h in freshly prepared DAB staining solution [1 mg/ml DAB (Beijing Dingguo Changsheng Biotechnology Co., Ltd., JD091) which was dissolved in 10 mM Na_2_HPO_4_ supplemented with 0.1% (v/v) Tween-20] and then washed with 70% ethanol to remove chlorophyll. The leaves were analyzed and photographed using a Nikon microscope (SMZ25; Nikon). The relative levels of DAB staining were quantitatively analyzed by using Photoshop CS6 software (Adobe). For superoxide anion labeling, seedlings were vacuum infiltrated with 0.1 mg/ml NBT (Sigma-Aldrich, N6876) in 25 mM HEPES buffer (pH 7.6) for 2 h in the dark. Seventy percent ethanol was used to remove chlorophyll from the leaves, then these leaves were imaged using a differential interference contrast (DIC) optical system (BX64; Olympus) and a charge-coupled device (CCD) camera (DP72; Olympus).

### Detection of H_2_O_2_ and $O_{2}^{•-}$ contents

The POD-coupled assay was utilized to quantify the H_2_O_2_ levels as previously reported (Yuan et al., 2017). First, 2 ml of HClO_4_ (1 M) containing insoluble polyvinylpyrrolidone (5%) was used to extract 0.2 g of the Arabidopsis seedlings. The homogenate was centrifuged at 12,000 g for 10 min, and the supernatant was neutralized to pH 5.6 with 5 M K_2_CO_3_ to pH 5.6 in the presence of 100 μl of 0.3 M phosphate buffer, pH 5.6. The solution was centrifuged for 1 min at 12,000 g, and the supernatant was then mixed with 1 unit of ascorbate oxidase and left to stand for 10 min at 25°C. 3-(Dimethylamino)benzoic acid (3.3 mM), 0.07 mM 3-methyl-2-benzothiazoline hydrazone, and 0.1 M phosphate buffer (pH 6.5) were added to 500 μl of the reaction mixture to start the final reaction. After standing for 30 min at 25°C, the 590-nm absorbance change in the solution was determined. A superoxide anion content detection kit (Beijing Solarbio Science and Technology Co., Ltd., BC1290) was used to measure the $O_{2}^{•-}$ contents according to the provided instructions.

### Determination of catalase, ascorbate peroxidase, and superoxide dismutase activities

Total CAT, APX, and SOD activities were analyzed using a Catalase Assay Kit (Beyotime Biotechnology), Ascorbate Peroxidase Assay Kit (Gelatins), and a Total Superoxide Dismutase Assay Kit with NBT (Beyotime Biotechnology), respectively, following supplied protocols. The CAT activity was assayed based on decreases in H_2_O_2_ accumulation. The APX activity was measured by calculating the AsA oxidation rate. The SOD activity was detected by NBT photoreduction inhibition.

### Western blot analysis

Total proteins were extracted using the plant protein extracting buffer containing 375 mM NaCl, 2.5 mM EDTA, 1% β-mercaptoethanol, 125 mM Tris–HCl (pH8.0), and 1% SDS. Protein concentrations were assayed using a bicinchoninic acid assay (BCA) protein assay kit (Beijing Dingguo Changsheng Biotechnology Co., Ltd.). Afterward, the proteins were separated by electrophoresis using 12% SDS-PAGE gel. Immunoblotting was performed on PVDF membranes with anti-HSC70 (Agrisera, AS08348) and anti-ACTIN (Abmart, M20009M). The intensity of each immunodetection band was determined using an image-processing and analysis software package (ImageJ, version 1.52v). Relative protein levels were normalized against those in control, which were set to 1.

## Results

### Osmotic stress promotes the expression of *cpHSC70-1*

**Figure 1:**
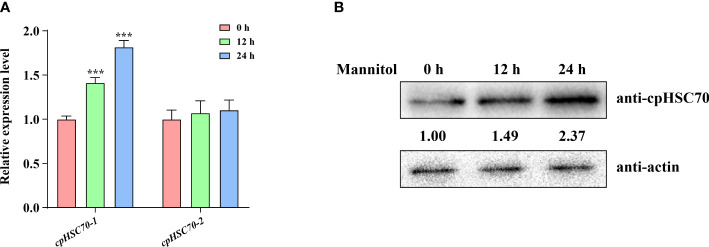
Osmotic stress induces *cpHSC70-1* expression. **(A)** The *cpHSC70-1* and *cpHSC70-2* expression levels of 5-day-old wild-type seedlings subjected to osmotic stress (300 mM mannitol for 6, 12 h) and its control. The data are means ( ± SEM) from at least three independent experiments. Asterisks indicate significant differences revealed using a Student’s *t*-test (***p < 0.001). **(B)** cpHSC70 protein level of 5-day-old wild-type seedlings treated with 300 mM mannitol for 0, 12, and 24 h. The intensity of each immunodetection band was measured by using an image-processing and analysis software package (ImageJ). The protein levels of 0 h were set to 1. Actin was used as control.

HSP70s play key roles in ensuring cellular homeostasis, whether cells are in normal or stressful environments (Leng et al., 2017). Although their family members, *cpHSC70s*, have been reported to be involved in some biotic stresses such as heat and drought (Su and Li, 2008; Latijnhouwers et al., 2010), the function of *cpHSC70s* in osmotic stress remains unknown. To investigate whether *cpHSC70s* are involved in plants osmotic stress response, we examined whether mannitol treatment affects the expression of *cpHSC70-1* and *cpHSC70-2*. Our reverse transcription PCR (RT-qPCR) analyses showed that osmotic stress significantly induced the expression of *cpHSC70-1*, but not *cpHSC70-2* (**Figure 1A**). Moreover, we measured cpHSC70 proteins in mannitol-treated wild-type plants; our Western blot analyses indicated that the mannitol-treated wild-type seedlings had higher cpHSC70 accumulation than untreated control (**Figure 1B**). Thus, our data suggest that osmotic stress promotes *cpHSC70-1* expression in the stressed plant.

### The *cpHSC70-1* functions positively in plant response to osmotic stress

**Figure 2:**
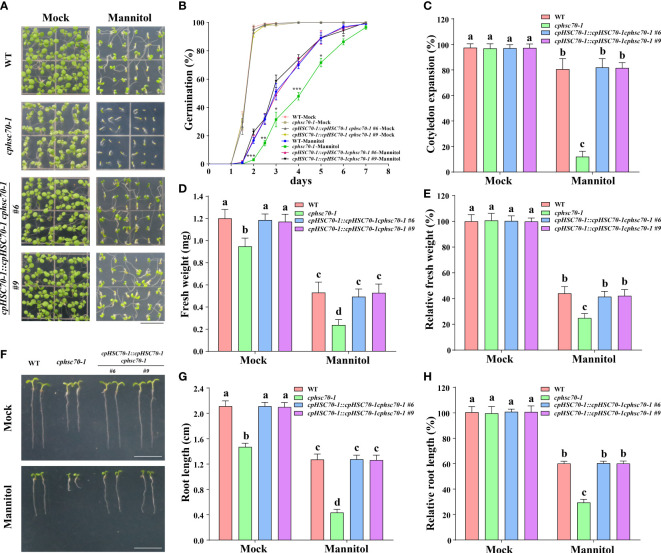
Loss-of-function mutation of *cpHSC70-1* increases plant sensitivity to osmotic stress. **(A)** Images of 5-day-old wild-type, *cphsc70-1*, and *cpHSC70-1::cpHSC70-1 cphsc70-1* seedlings grown on 1/2 MS medium with or without (Mock) 300 mM mannitol. Bars = 1 cm. **(B)** Germination rate of the wild-type, *cphsc70-1*, and *cpHSC70-1::cpHSC70-1 cphsc70-1* plants in response to 300 mM mannitol. **(C)** Rate of cotyledon expansion, **(D)** fresh weight, and **(E)** relative fresh weight of the wild-type, *cphsc70-1*, and *cpHSC70-1::cpHSC70-1 cphsc70-1* plants in panel **(A)**. **(F)** Images of 5-day-old wild-type, *cphsc70-1*, and *cpHSC70-1::cpHSC70-1 cphsc70-1* seedlings grown on 1/2 MS medium with or without (Mock) 300 mM mannitol. Bars = 1 cm. **(G)** Root length and **(H)** relative root length of the plants shown in **(F)**. The data are means ( ± SD) from at least three independent experiments (n ≥60 for germination rate and n ≥30 for root length). Asterisks indicate significant differences revealed using a Student’s *t*-test (*p < 0.05; **p < 0.01; ***p< 0.001). Different letters indicate significant differences as determined using ANOVA followed by Tukey’s test (*P* < 0.05).

The induction of *cpHSC70-1* expression by osmotic stress as shown in our above results implies a possible role of this gene in plant response to osmotic stress. To verify this possibility, we obtained the mutant *cphsc70-1* (SALK_140810), in which T-DNA was inserted in the second intron of *cpHSC70-1* and *cpHSC70-1* expression was significantly reduced in the mutant (**Supplementary Figures 1A, B**) and generated the complementation lines (*cpHSC70-1::cpHSC70-1 cphsc70-1*) by expressing the full-length coding sequence of *cpHSC70-1* driven under its native promoter in *cphsc70-1* mutant (**Supplementary Figure 1B**). Then, we examined the phenotypes of the mutant and its complementation lines under osmotic stress. We found that when grown under normal conditions, *cphsc70-1* exhibited variegated cotyledons, malformed leaves, small size, and impaired root growth compared with the wild type as previously reported (Su and Li, 2008; Chu et al., 2020), but *cphsc70-1* plants showed a similar germination rate and cotyledon expansion rate as the wild type had (**Figures 2A-C**). When challenged with osmotic stress, the *cphsc70-1* seedlings had a reduced germination rate and cotyledon expansion rate than wild-type seedlings (**Figures 2B, C**). In addition, the fresh weight and primary root elongation were further repressed by osmotic stress in the mutant compared with the wild type (**Figures 2D, H**). Regarding the *cpHSC70-1::cpHSC70-1 cphsc70-1* plants, they behaved as the wild type under either normal or stressed conditions (**Figures 2A-H**). Taken together, our results indicate that *cpHSC70-1* functions positively in plant response to osmotic stress.

### Overexpression of *cpHSC70-1* enhances plant tolerance to osmotic stress

**Figure 3:**
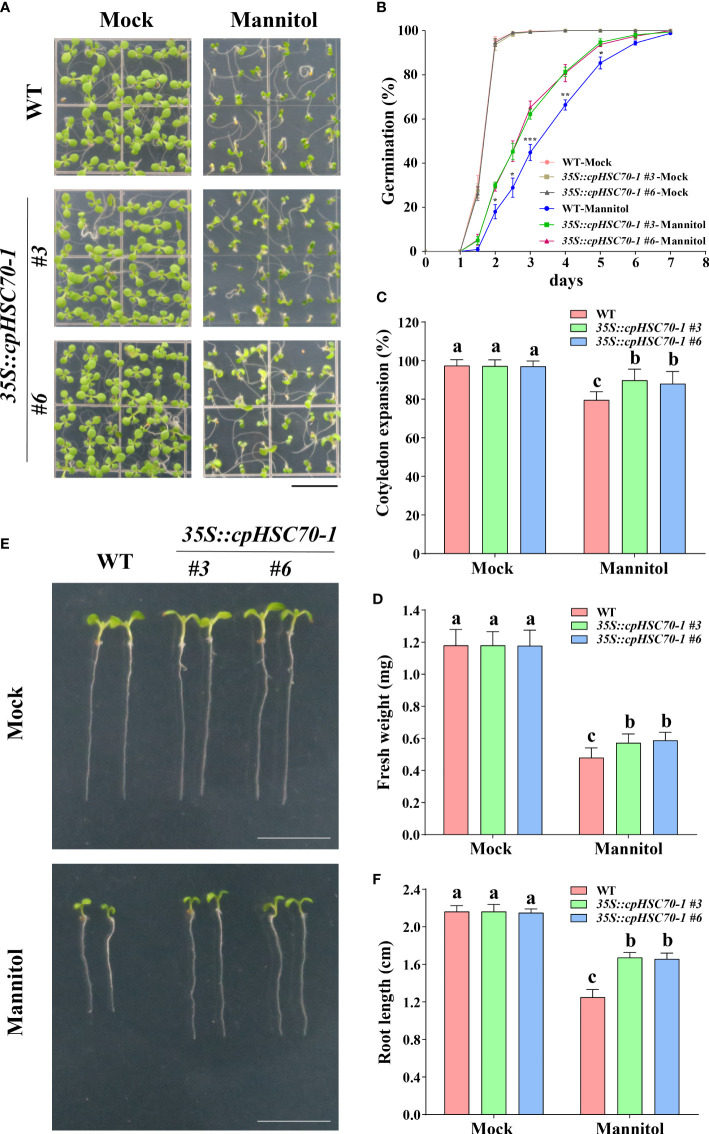
Overexpression of *cpHSC70-1* enhanced osmotic stress tolerance. **(A)** Images of 5-day-old wild-type and *35S::cpHSC70-1* seedlings grown on 1/2 MS medium with or without (Mock) 300 mM mannitol. Bars = 1 cm. **(B)** Germination rate of the wild-type and *35S::cpHSC70-1* plants in response to 300 mM mannitol. **(C)** Rate of cotyledon expansion and **(D)** fresh weight of wild-type and *35S::cpHSC70-1* plants in panel **(A)**. **(E)** Images of 5-day-old wild-type and *35S::cpHSC70-1* seedlings grown on 1/2 MS medium with or without (Mock) 300 mM mannitol. Bars = 1 cm. **(F)** Root length of the plants shown in **(E)**. The data are means ( ± SD) from at least three independent experiments (n ≥60 for germination rate and n ≥ 30 for root length). Asterisks indicate significant differences revealed using a Student’s *t*-test (* *p* < 0.05; **p < 0.01; ***p < 0.001). Different letters indicate significant differences as determined using ANOVA followed by Tukey’s test (*p* < 0.05).

Further, we tested whether overexpression of this gene can confer plants more tolerance to osmotic stress. For this end, we generated transgenic lines *35S::cpHSC70-1* by overexpressing *cpHSC70-1* under the control of the 35S promoter in the Col-0 background Arabidopsis. RT-qPCR results showed that the *cpHSC70-1* expression in the overexpression lines was higher than that in the wild type (**Supplementary Figure 1B**). While *35S::cpHSC70-1* plants showed no aberrant phenotype compared to the wild type under normal growth conditions (**Figure 3**), these seedlings had a higher germination rate, promoted the cotyledon expansion rate, and increased fresh weight and longer primary root length than the wild-type seedlings (**Figures 3A-F**). These findings reveal that overexpression of *cpHSC70-1* enhances osmotic stress tolerance in the transgenic plants.

### *cpHSC70-1* regulates the expression of the genes involved in plant stress response

**Figure 4:**
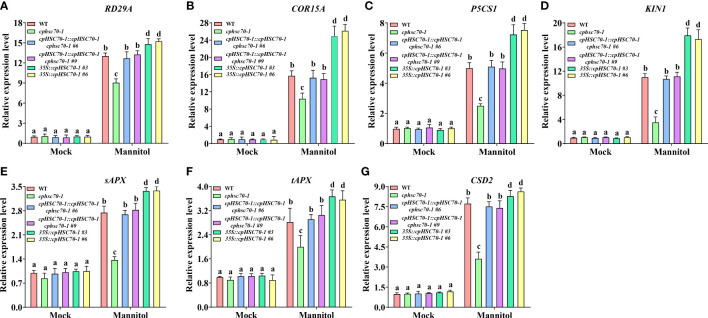
Effects of cpHSC70-1 on expression of genes involved in stress response and ROS-scavenging system. **(A–G)** The expression levels of **(A)** *RD29A*, **(B)** *COR15A*, **(C)** *P5CS1*, **(D)** *KIN1*, **(E)** *sAPX*, **(F)** *tAPX*, and **(G)** *CSD2* of the 5-day-old wild-type, *cphsc70-1*, *cpHSC70-1::cpHSC70-1 cphsc70-1*, and *35S::cpHSC70-1* plants treated with or without (Mock) 300 mM mannitol for 12 h. The expression of these genes was determined by RT-qPCR and correlated with that of the WT, the value of which was set as 1. *ACTIN2* was used as the reference gene. The data are means ( ± SEM) from at least three independent experiments. Different letters indicate significant differences as determined using ANOVA followed by Tukey’s test (*p* < 0.05).

Previous studies reported that various abiotic stresses including high salinity and osmotic stress modulate the expression of stress-responsive genes (Wang et al., 2021a; Ding et al., 2022). To explore the impact of *cpHSC70-1* on the molecular basis of osmotic stress response, we detected the transcripts of several key genes involved in osmotic stress (*RD29A*, *KIN1*, *COR15A*, and *P5CS1*) (Wang et al., 2021b). We found that the transcripts of all tested genes were similar in *cphsc70-1*, *35S::cpHSC70-1*, *cpHSC70-1::cpHSC70-1 cphsc70-1*, and wild-type plants under normal conditions (**Figures 4A-D**). However, upon osmotic stress, *phsc70-1* plants suppressed but *35S::cpHSC70-1* plants promoted the expression of these genes compared with both wild-type and *cpHSC70-1::cpHSC70-1 cphsc70-1* plants (**Figures 4A-D**). These results suggest that the expression of the stress-responsive genes could be involved in *cpHSC70-1*-mediated plant osmotic stress response.

### The *cpHSC70-1* regulates ROS homeostasis of plants under osmotic stress

**Figure 5:**
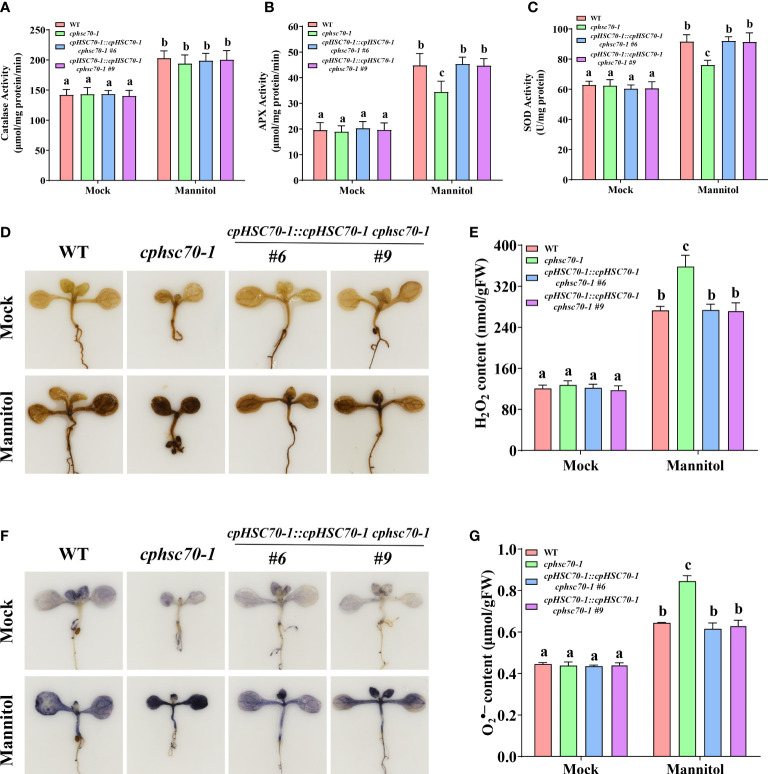
The *cphsc70-1* mutant has higher ROS accumulation and reduced APX and SOD activity. **(A)** Catalase activity, **(B)** APX activity, and **(C)** SOD activity of the 5-day-old wild-type, *cphsc70-1*, and *cpHSC70-1::cpHSC70-1 cphsc70-1* plants treated with or without (Mock) 300 mM mannitol for 2 days. **(D)** The DAB-staining images of leaves from the 5-day-old wild-type, *cphsc70-1*, and *cpHSC70-1::cpHSC70-1 cphsc70-1* plants treated with or without (Mock) 300 mM mannitol for 2 days. **(E)** Measurements of H_2_O_2_ in the 5-day-old wild-type, *cphsc70-1*, and *cpHSC70-1::cpHSC70-1 cphsc70-1* plants treated with or without (Mock) 300 mM mannitol for 2 days. **(F)** The NBT-staining images of leaves from the 5-day-old wild-type, *cphsc70-1*, and *cpHSC70-1::cpHSC70-1 cphsc70-1* plants treated with or without (Mock) 300 mM mannitol for 2 days. **(G)** Measurements of $O_{2}^{•-}$ in the 5-day-old wild-type, *cphsc70-1*, and *cpHSC70-1::cpHSC70-1 cphsc70-1* plants treated with or without (Mock) 300 mM mannitol for 2 days. The data are means ( ± SD) from at least three independent experiments. Different letters indicate significant differences as determined using ANOVA followed by Tukey’s test (*p* < 0.05).

It is reported that ROS is involved in plant response to osmotic stress (Foyer and Noctor, 2005); (Koussevitzky et al., 2008; Vishwakarma et al., 2015; Li et al., 2019; Wang et al., 2021a). Thus, we also tested the expression of genes involved in the ROS-scavenging system (*CAT1*, *CAT2*, *CAT3*; *APX1*, *sAPX*, *tAPX*; *CSD1*, *CSD2*, *CSD3*) in *cphsc70-1* and *35S::cpHSC70-1* plants under osmotic stress. Our results showed that while *cphsc70-1*, *35S::cpHSC70-1*, *cpHSC70-1::cpHSC70-1 cphsc70-1*, and wild-type plants had similar expressions of *CAT1*, *CAT2*, *CAT3*, *APX1*, *CSD1*, and *CSD3*, the *cphsc70-1* mutant reduced but *cpHSC70-1* overexpression lines increased the expression of *sAPX*, *tAPX*, and *CSD2* compared with *cpHSC70-1::cpHSC70-1 cphsc70-1* and wild-type plants when subjected with mannitol treatment (**Figures 4E-G**; **Supplementary Figures 2A-F**). Consistently, all tested plants had similar CAT activity when grown under both normal and stressful conditions; however, osmotic stress-promoted activities of APX and SOD in both *cpHSC70-1::cpHSC70-1 cphsc70-1* and wild-type plants were repressed in *cphsc70-1* mutant but enhanced in *35S::cpHSC70-1* plants (**Figures 5A-C**; **Supplementary Figures 3A-C**). These results suggest the possible involvement of ROS homeostasis in *cpHSC70-1*-mediated plant osmotic stress response. Therefore, we examined the contents of H_2_O_2_ in plant response to osmotic stress by using both 3,3-diaminobenzidine (DAB) staining experiments and the POD-coupled assay. Our results showed that while the stress elevated H_2_O_2_ levels in the stressed *cphsc70-1*, *35S::cpHSC70-1, cpHSC70-1::cpHSC70-1 cphsc70-1* and wild-type plants compared with their untreated control, respectively, H_2_O_2_ accumulation was higher in *cphsc70-1* but lower in *35S::cpHSC70-1* than that in *cpHSC70-1::cpHSC70-1 cphsc70-1* and the wild type upon osmotic stress treatment (**Figures 5D, E**; **Supplementary Figures 3D, E**). Similar results were found when we assayed the abundance of the superoxide anion $\left(O_{2}^{•-}\right)$ using NBT staining and spectrophotometry (**Figures 5F, G**; **Supplementary Figures 3F, G**). These findings indicate that *cpHSC70-1* regulates plant ROS homeostasis under osmotic stress.

## Discussion

HSP70 proteins are widespread and play key roles in organisms ranging from prokaryotes to land plants. They have been mainly reported in heat shock responses, protein folding and translocation, and prevention of protein aggregation (Miemyk, 1997). However, whether the chloroplast-located cpHSC70-1 participates in plant response to osmotic stress remains unknown. Here, we provide evidence that *cpHSC70-1* is required for plant tolerance to osmotic stress because *cphsc70-1* plants have higher sensitivity to osmotic stress and *35S::cpHSC70-1* plants exhibit higher osmotic stress tolerance.

It has been documented that there are two chloroplast-localized HSPs (cpHSC70-1 and cpHSC70-2) in Arabidopsis. These two proteins are structurally homologous based on their protein sequence alignment and play important roles in chloroplast development and functional integrity (Sung et al., 2001; Latijnhouwers et al., 2010). In this study, we found that osmotic stress induces the expression of *cpHSC70-1*, but the *cpHSC70-2* expression is not affected by the stress (**Figure 1**). Thus, we also examined whether *cpHSC70-2* is involved in plant response to osmotic stress. We obtained the mutant *cphsc70-2* (SALK_095715), in which T-DNA was inserted in the second intron of *cpHSC70-2* and *cpHSC70-2* expression was significantly reduced in the mutant (**Supplementary Figures 1C, D**), and we assayed the stress response of this mutant under osmotic stress. We found that *cphsc70-2* and wild-type plants had no substantial distinction in terms of germination rate, cotyledon expansion rate, fresh weight, and primary root elongation under either normal or osmotic-stressed conditions (**Supplementary Figure 4**). Further, the transgenic *35S::cpHSC70-2* lines, in which *pHSC70-2* is overexpressed under the control of the 35S promoter (**Supplementary Figure 1D**), does not show higher stress tolerance compared with the wild type (**Supplementary Figure 4**). Thus, our data do not support the involvement of *cpHSC70-2* in plant response to osmotic stress. Similar observations were also reported when the role of *cpHSC70-1/2* was examined in plant response to drought and heat stresses (Su and Li, 2008; Latijnhouwers et al., 2010). These studies indicate that *cpHSC70-1* acts positively in plant response to these stresses, but *cpHSC70-2* is not required for plant tolerance to these stresses.

Many studies indicate that various abiotic stresses induce the expression of stress-responsive genes (Zhang et al., 2019; Fu et al., 2021; Ding et al., 2022). There have been various reports that hypersensitive mutants have increased the induction of stress-inducible genes. For example, the salt stress-induced expression of *COR15A* and *ABCG6* is enhanced in salt stress-sensitive *glyI2* mutants (Fu et al., 2021). Similarly, *dpg1* mutants have higher expressions of *RD29A* and *RD29B* than the wild type under high salinity (Yi et al., 2019), and *gcn20* mutants are more sensitive to salt stress with higher expressions of *KIN1*, *KIN2*, and *COR15A* (Ding et al., 2022). However, it is widely documented that hypersensitive mutants have a decreased induction of stress-inducible genes. For instance, *cand2-1* mutants are hypersensitive to osmotic stress with decreased induction of *RD29A*, *KIN1*, *P5CS1*, and *COR15A* (Wang et al., 2021b). Also, mutation of *DCD* results in the inhibition of cadmium stress-induced *PCR1* and *PDR8* (Zhang et al., 2020). A recent report documents that expression levels of *COR15A*, *COR47*, and *RD29A* are significantly lower in *rboh-D*, *rboh-F*, and *rboh-DF* mutants than in the WT when treated with cold, and these mutants are less tolerant to cold stress (Liu et al., 2022a). In addition, salt and drought stress-induced *RD29A* and *RD22* are significantly higher in the *amiR-TSB1* mutants than in the wild type when the mutants show higher tolerances to drought and salt stress than the wild type (Liu et al., 2022b). Our experimental results indicate that osmotic stress promoted the expression of stress-responsive genes such as *RD29A*, *COR15A*, *P5CS1*, and *KIN1* in wild-type plants which are repressed in *cphsc70-1* mutant but enhanced in *35S::cpHSC70-1* plants. However, how cpHSC70-1 modulates the expression of these genes is unclear. ABA is a central phytohormone regulating plant responses to osmotic stress (Tivendale et al., 2014; Julkowska and Testerink, 2015). Many ABA-inducible genes such as *RD29A*, *KIN1*, and *COR15A* function in various abiotic stresses including osmotic stress (Ding et al., 2022). We speculate that cpHSC70-1 may be involved in regulating the expression of these stress-responsive genes by affecting ABA accumulation or ABA signaling, which is worthy of further exploration. Additionally, it has been documented that cpHSC70-1 can interact with GENOMES UNCOUPLED1 (GUN1), a protein that participates in multiple retrograde signaling pathways, regulating the expression of many nuclear-encoded genes (Wu et al., 2019). Thus, investigating whether GUN1 functions in cpHSC70-1-mediated plant osmotic stress response by modulating the expression of nuclear-encoded stress-responsive genes could be a future research direction.

Various environmental stresses including osmotic stress, result in oxidative damages caused by overaccumulated reactive oxygen species (ROS) (Wenjing et al., 2020; Zhao et al., 2020; Wang et al., 2021b; Zhang et al., 2021). These ROS can affect proteins, lipids, and nucleic acids if accumulated over a certain threshold, leading to cell damage and death (Yuan et al., 2017; Nadarajah, 2020). Thus, reducing stress-induced ROS overaccumulation is one of the most important and common protective mechanisms for plants under an adverse environment. Our study shows that the knockout of *cpHSC70-1* has decreased APX and SOD activities and increased ROS accumulation, and overexpression of *cpHSC70-1* in wild-type lines has higher ROS detoxification capacity and less ROS. It is known that a system of posttranslational protein transport into the chloroplast is absolutely essential for its functions such as photosynthesis, and cpHSC70-1 is a motor for protein import into the chloroplast and its mutation significantly reduces the efficiency of protein import (Su and Li, 2010; Li et al., 2020). In animals, HSP70 modulates SOD2 activity by promoting the import of SOD2 into the mitochondria (Zemanovic et al., 2018). We also notice that the mutation of *cpHSC70-1* reduces activities of APX and SOD, but not CAT, under osmotic stress. It is possible that cpHSC70-1 may regulate the import of sAPX, tAPX, and CSD2 into the chloroplast, but not CAT1, CAT2, and CAT3, into the peroxisome, resulting in changes in the activities of APX and SOD under osmotic stress. Chloroplasts are thought to be the main source and target of ROS (Waszczak et al., 2018); thus, we speculate that when plants are subjected to osmotic stress, this impaired import of sAPX, tAPX, and CSD2 into the chloroplast in *cphsc70-1* mutants, resulting in reduced activities of APX and SOD, which in turn causes ROS accumulation. Further experiments are needed to verify this hypothesis.

## Data availability statement

The original contributions presented in the study are included in the article/**Supplementary Material**. Further inquiries can be directed to the corresponding author.

## Author contributions

FD and BZ conceived and designed the project. FD and FL performed the experiments. FD and BZ analyzed the data and wrote the manuscript. All authors contributed to the article and approved the submitted version.

## Acknowledgments

We thank Prof. Ying-Tang Lu (Wuhan University, China) for providing guidance and valuable advice.

## Conflict of interest

The authors declare that the research was conducted in the absence of any commercial or financial relationships that could be construed as a potential conflict of interest.

## Publisher’s note

All claims expressed in this article are solely those of the authors and do not necessarily represent those of their affiliated organizations, or those of the publisher, the editors and the reviewers. Any product that may be evaluated in this article, or claim that may be made by its manufacturer, is not guaranteed or endorsed by the publisher.
