# Supplementary material for: A plastid-targeted heat shock cognate 70-kDa protein confers osmotic stress tolerance by enhancing ROS scavenging capability
Source: Front Plant Sci. 2022 Oct 5;13:1012145. doi: 10.3389/fpls.2022.1012145 (PMC9581120; doi:10.3389/fpls.2022.1012145)
Supplement: Supplementary file 1 [file DataSheet_1.doc]

**
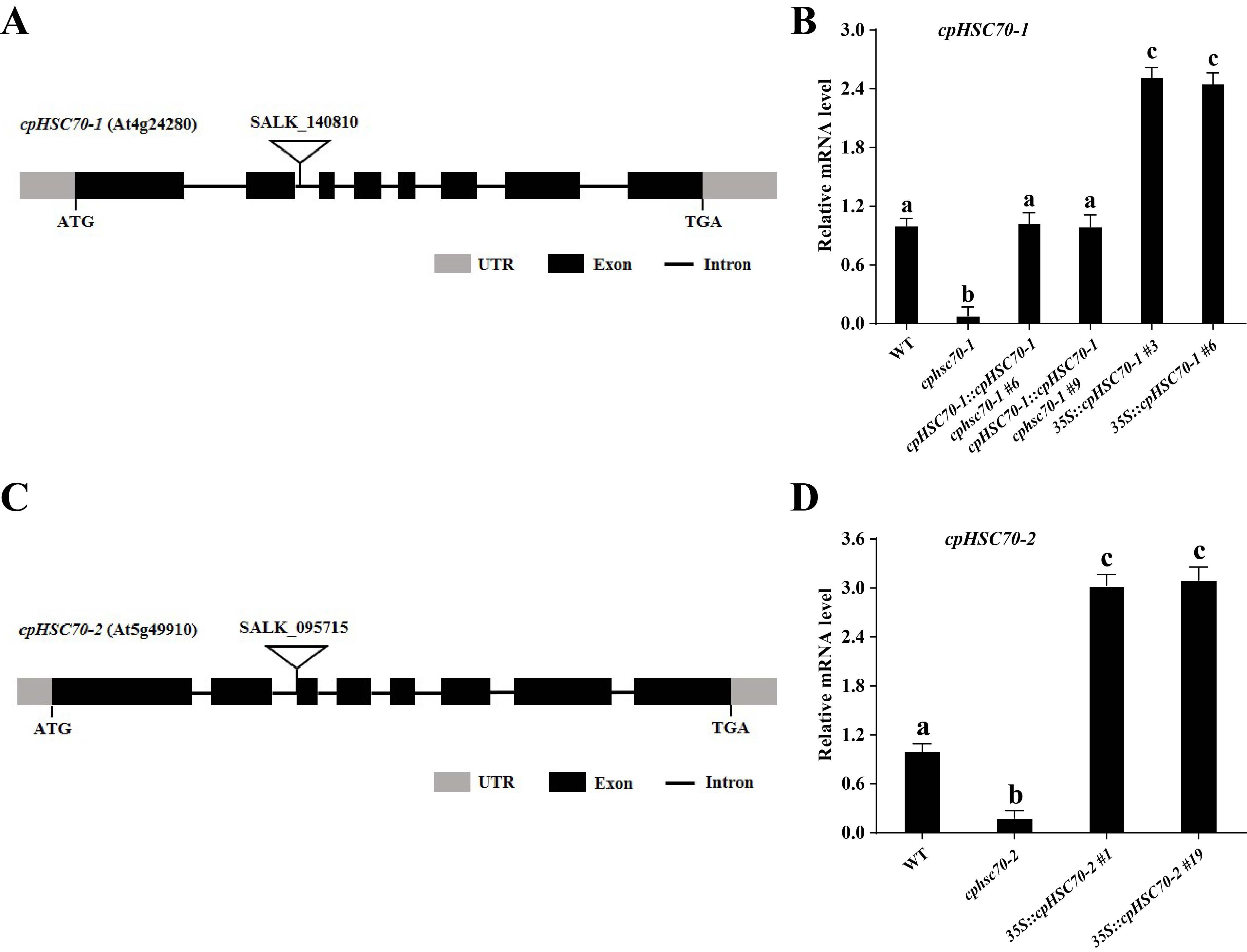
**

**SUPPLEMENTARY** **FIGURE** **1** **|** The identification of mutants and transgenic plants. **(A)** The diagram of the *cpHSC70-1* gene with T-DNA insertion site. Rectangles represent exons and lines between the exons stand for introns; Flag indicate T-DNA insertion site. **(B)** Expression analysis of *cpHSC70-1* gene in the wild-type, *cphsc70-1*, *cpHSC70-1::cpHSC70-1 cphsc70-1*, and *35S::cpHSC70-1* plants, the *ACTIN2* gene was used as an internal control. **(C)** The diagram of the *cpHSC70-2* gene with T-DNA insertion site. Rectangles represent exons and lines between the exons stand for introns; Flag indicate T-DNA insertion site. **(D)** Expression analysis of *cpHSC70-2* gene in the wild-type, *cphsc70-2*, and *35S::cpHSC70-2* plants, the *ACTIN2* gene was used as an internal control. The expression of these two genes was determined by RT-qPCR and correlated to that of the WT, the value of which was set as 1. The data are means (±SEM) from at least three independent experiments. Different letters indicate significant differences as determined using ANOVA followed by Tukey’s test (*P* < 0.05).

**
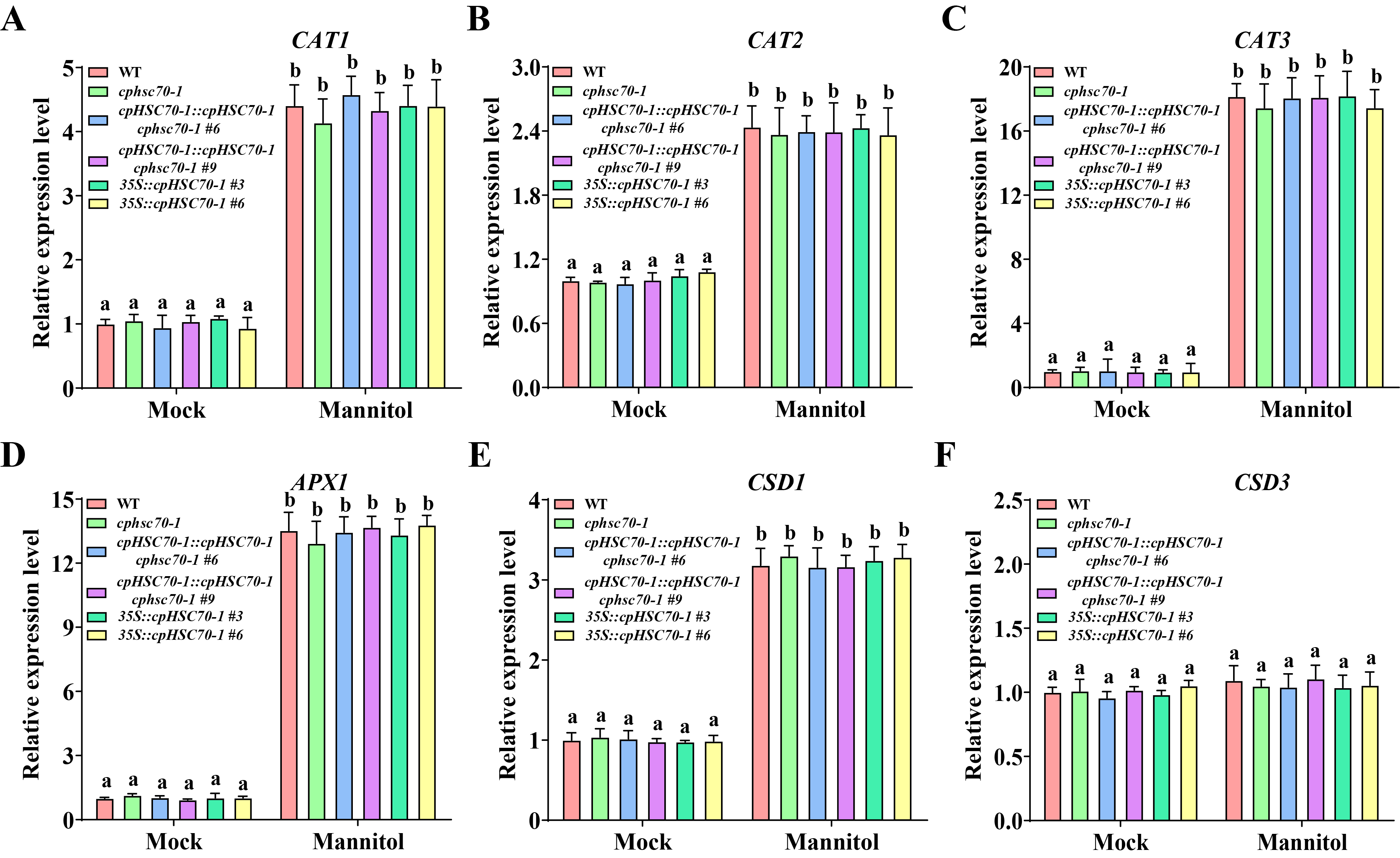
**

**SUPPLEMENTARY** **FIGURE** **2** **|** Effects of cpHSC70-1 on expression of ROS-scavenging-related genes. **(A**–**F)** The expression levels of **(A)** *CAT1*, **(B)** *CAT2*, **(C)** *CAT3*, **(D)** *APX1*, **(E)** *CSD1*, and **(F)** *CDS3* of 5-day-old the wild-type, *cphsc70-1*, *cpHSC70-1::cpHSC70-1 cphsc70-1*, and *35S::cpHSC70-1* plants treated with or without (Mock) 300 mM mannitol for 12 h. The expression of these genes was determined by RT-qPCR and correlated to that of the WT, the value of which was set as 1. *ACTIN2* was used as the reference gene. The data are means (±SEM) from at least three independent experiments. Different letters indicate significant differences as determined using ANOVA followed by Tukey’s test (*P* < 0.05).

**
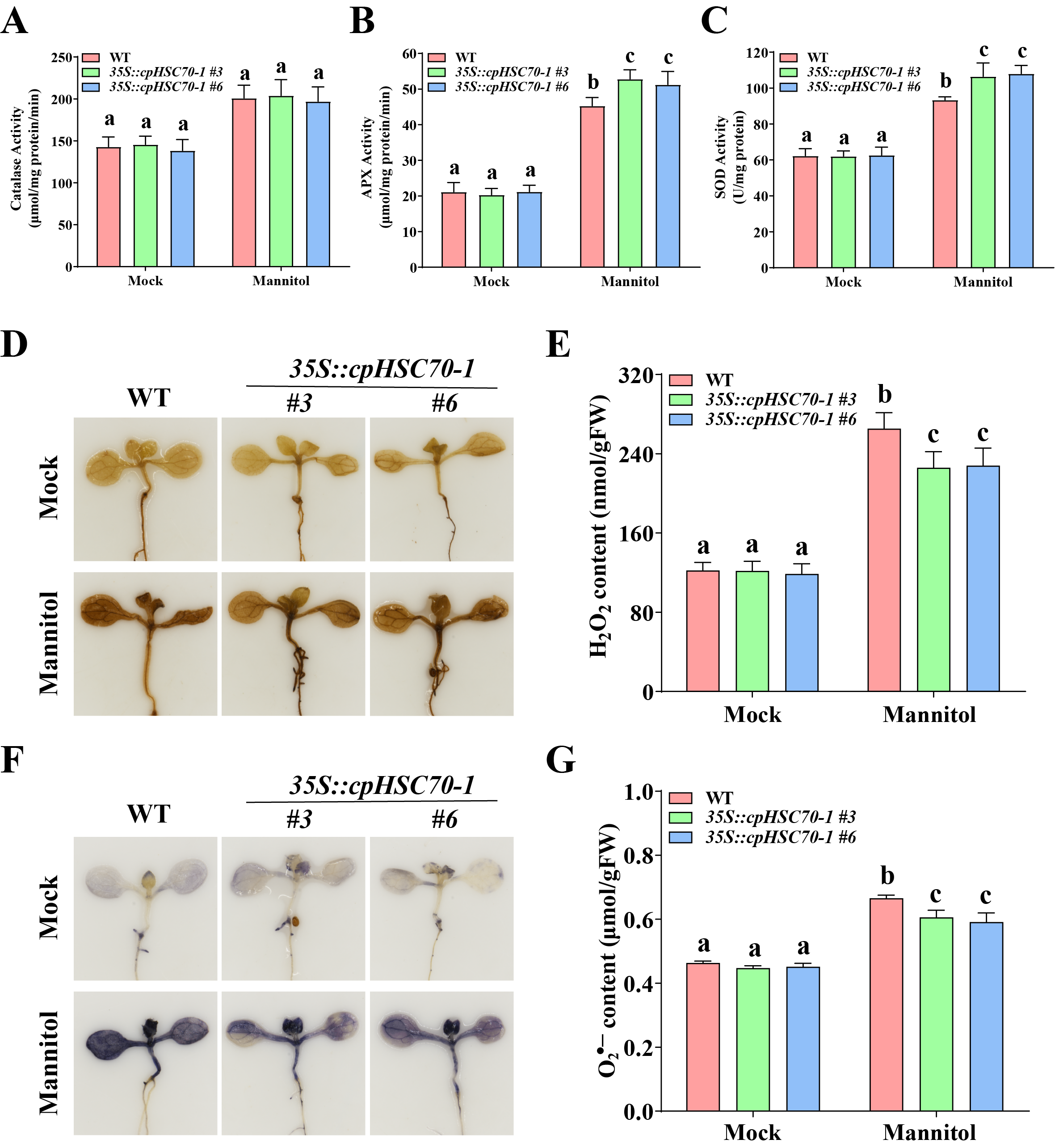
**

**SUPPLEMENTARY** **FIGURE** **3** **|** The *35S::cpHSC70-1* plants have less ROS accumulation and increased APX and SOD activity under osmotic tress. **(A)** Catalase activity, **(B)** APX activity, and **(C)** SOD activity of the 5-day-old wild-type, and *35S::cpHSC70-1* plants treated with or without (Mock) 300 mM mannitol for 2 days. **(D)** The DAB-staining images of leaves from the 5-day-old wild-type, and *35S::cpHSC70-1* plants treated with or without (Mock) 300 mM mannitol for 2 days. **(E)** Measurements of H_2_O_2_ in the 5-day-old wild-type, and *35S::cpHSC70-1* plants treated with or without (Mock) 300 mM mannitol for 2 days. **(F)** The NBT-staining images of leaves from the 5-day-old wild-type, and *35S::cpHSC70-1* plants treated with or without (Mock) 300 mM mannitol for 2 days. **(G)** Measurements of O_2_^•–^ in the 5-day-old wild-type, and *35S::cpHSC70-1* plants treated with or without (Mock) 300 mM mannitol for 2 days. The data are means (±SD) from at least three independent experiments. Different letters indicate significant differences as determined using ANOVA followed by Tukey’s test (*P* < 0.05).

**
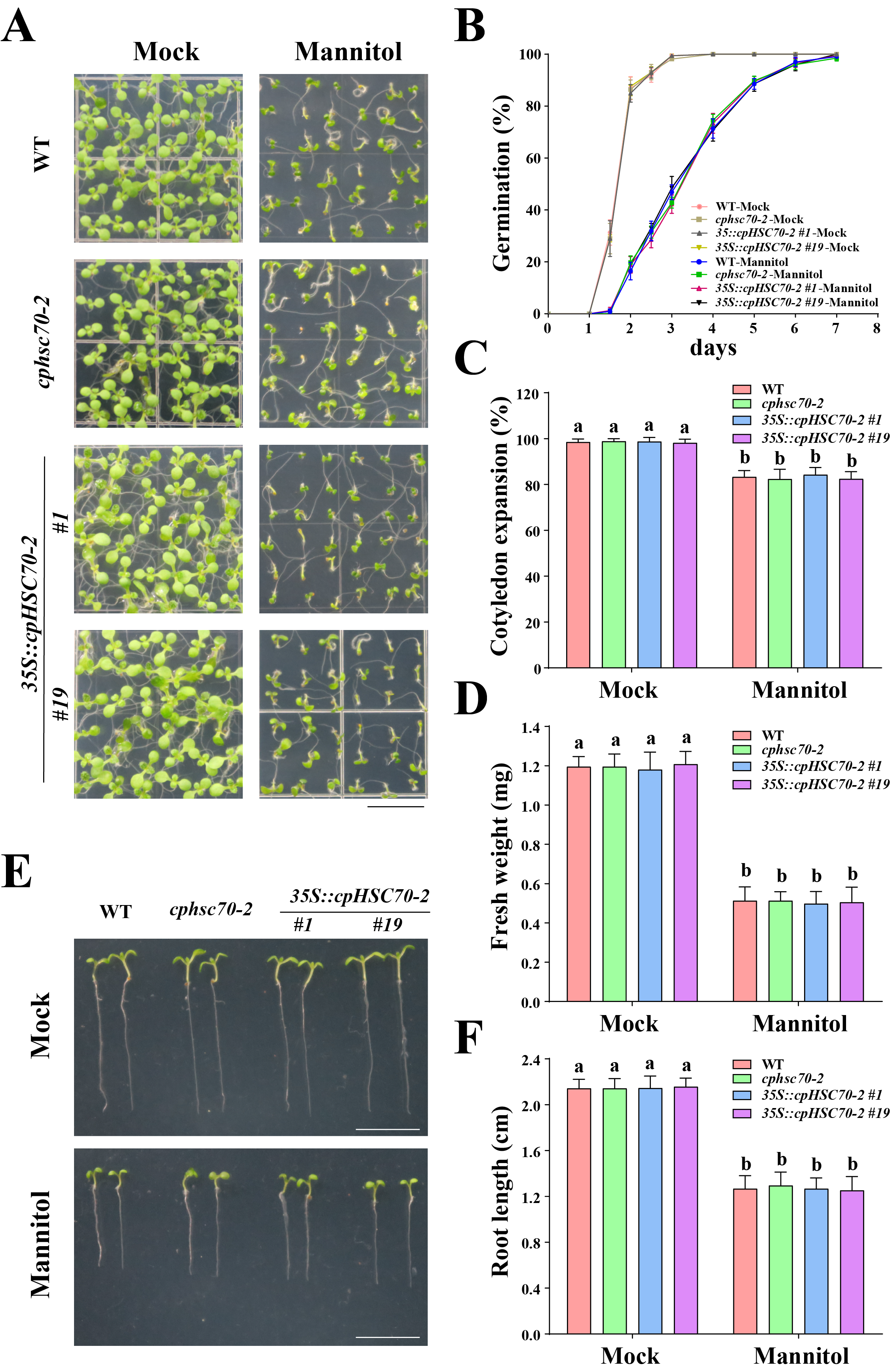
**

**SUPPLEMENTARY** **FIGURE** **4** **|** Phenotypic analysis of the *cphsc70-2* and *35S::cpHSC70-2* plants under osmotic stress. **(A)** Images of 5-day-old wild-type, *cphsc70-2*, and *35S::cpHSC70-2* seedlings grown on 1/2 MS medium with or without (Mock) 300 mM mannitol. Bars = 1 cm. **(B)** Germination rate of the wild-type and *35S::cpHSC70-1* plants in response to 300 mM mannitol. **(C)** Rate of cotyledon expansion and **(D)** fresh weight of wild-type, *cphsc70-2*, and *35S::cpHSC70-2* plants in panel **(A)**. **(E)** Images of 5-day-old wild-type and *35S::cpHSC70-1* seedlings grown on 1/2 MS medium with or without (Mock) 300 mM mannitol. Bars = 1 cm. **(F)** Root length of the plants shown in **(E)**. The data are means (±SD) from at least three independent experiments (n≥60 for germination rate and n≥30 for root length). Different letters indicate significant differences as determined using ANOVA followed by Tukey’s test (*P* < 0.05).

**Supplemental Table 1. List of all primers used in this study.**

| **Primer name** | **Sequence (5'-3')** |
| --- | --- |
| **Genomic DNA PCR** | |
| Salk_140810 LP | GATTCACAGAGGACAGCTAC |
| Salk_140810 RP | TGAATCTCCTGATGAAGCAC |
| Salk_095715 LP | GTCCTTTCACTACTCCAACC |
| Salk_095715 RP | CTGAGGATGATGTGTCAGAC |
| LBb1.3 | ATTTTGCCGATTTCGGAAC |
| LBb1 | GCGTGGACCGCTTGCTGCAACT |
| **RT-qPCR** | |
| *ACTIN2* RT-F | CTTGCACCAAGCAGCATGAA |
| *ACTIN2* RT-R | CCGATCCAGACACTGTACTTCCTT |
| *cpHSC70-1* RT-F | ACTGTGCCTGCTTACTTCAA |
| *cpHSC70-1* RT-R | ACAATGTCACTCACATCTCC |
| *cpHSC70-2* RT-F | GTCCTTTCACTACTCCAACC |
| *cpHSC70-2* RT-R | CTGAGGATGATGTGTCAGAC |
| *RD29A* RT-F | GCCGACGGGATTTGACG |
| *RD29A* RT-R | GCCGACGGGATTTGACG |
| *COR15A RT-F* | ACCGCAGATACATTGGGTAAA |
| *COR15A* RT-R | GTTTGCGGCTTCTTTTCCT |
| *P5CS1* RT-F | TGTGTGTGGTTTTGGTAGACG |
| *P5CS1* RT-R | GCAAAAGCACGTGAACGAT |
| *KIN1* RT-F | TCTCATCATCACTAACCAAAAC |
| *KIN1* RT-R | GACCCGAATCGCTACTTG |
| *APX1* RT-F | TTTCCACCCTGGAAGAGAGGAC |
| *APX1* RT-R | TCACAACCCTTGGTAGCATCAGG |
| *sAPX* RT-F | AGAATGGGATTAGATGACAAGGAC |
| *sAPX* RT-R | TCCTTCTTTCGTGTACTTCGT |
| *tAPX* RT-F | GCTAGTGCCACAGCAATAGAGGAG |
| *tAPX* RT-R | TGATCAGCTGGTGAAGGAGGTC |
| *CAT1* RT-F | TGGGATTCAGACAGGCAAGAACG |
| *CAT1* RT-R | GTTTGGCCTCACGTTAAGACGAGT |
| *CAT2* RT-F | TCAAACCATGGATCCTTACAAGT |
| *CAT2* RT-R | TGTTCCATACAGGAGCACCA |
| *CAT3* RT-F | AAGCCTATTTGGGGGATCAT |
| *CAT3* RT-R | TTGTACGCGCTTGAAGGAC |
| *CSD1* RT-F | TGAACTCAGCCTGGCTACTGG |
| *CSD1* RT-R | AGCCACACACCAGAAGATACACAC |
| *CSD2* RT-F | CGTCTTCTCATTCCTCCTTCC |
| *CSD2* RT-R | GGGTTGAAATGTGGTCCTGTT |
| *CSD3* RT-F | CTGCGTTTCCAGTTGATTTGCT |
| *CSD3* RT-R | GGCGATTTGGGTAACATTCTTG |
| **Molecular Cloning** | |
| *cpHSC70-1*-promoter-pCAMBIA1300 F | CTCGGTACCCGGGGATCCATATATATATATATATAAATCATATAGTAACG |
| *cpHSC70-1*-promoter-pCAMBIA1300 R | GTCGACTCTAGAGGATCCTCATTGGCTGTCTGTGAAGT |
| *cpHSC70-1*-pCAMBIA1300S F | AAATCTATCTCTCTCGAGATGGCATCTTCAGCCGCCCA |
| *cpHSC70-1*-pCAMBIA1300S R | GTCCTTATAATCCTCGAGTTGGCTGTCTGTGAAGTCAG |
| *cpHSC70-2*-pCAMBIA1300S F | AAATCTATCTCTCTCGAGATGGCTTCCTCCGCCGCCCA |
| *cpHSC70-2*-pCAMBIA1300S R | GTCCTTATAATCCTCGAGATTGCTGTCTGTGAAGTCAG |
